# From Trials to Treatment: Current Evidence Supporting Faricimab Use in nAMD and DME

**DOI:** 10.3390/jcm15186956

**Published:** 2026-09-08

**Authors:** José María Ruiz-Moreno, Carolina Bernal-Morales, Olivia Esteban-Floría, Enrique Rodríguez-de-la-Rúa, Lidia Remolí-Sargues, Joaquín Borras-Blasco, Alba Gómez-Benlloch, Esther Cilveti, Sonia Valsero-Franco, Coral Arriola-Naharro, Paula García-Lunar, Sara Kaminski-Santamaría, Belén Muñoz-Molina, Alfredo García-Layana

**Affiliations:** 1 Department of Ophthalmology, Puerta de Hierro-Majadahonda University Hospital, 28222 Madrid, Spain; 2 Ocular Microsurgery Institute (IMO), 28035 Madrid, Spain; 3 Department of Ophthalmology, Castilla-La Mancha University, 02001 Albacete, Spain; 4 Institute of Ophthalmology, Hospital Clínic of Barcelona, 08036 Barcelona, Spain; carolbernalmo@gmail.com; 5 August Pi i Sunyer Biomedical Research Institute, 08036 Barcelona, Spain; 6 Department of Ophthalmology, University Clinic Hospital Lozano Blesa, 50009 Zaragoza, Spain; oliviaestebanfloria@hotmail.com; 7 Aragon Health Research Institute, 50009 Zaragoza, Spain; 8 Faculty of Medicine, University of Zaragoza, 50009 Zaragoza, Spain; 9 Department of Ophthalmology, University Hospital Virgen Macarena, 41009 Seville, Spain; erdelarua@gmail.com; 10 Department of Surgery, University of Seville, 41004 Seville, Spain; 11 Health Outcomes-Oriented Cooperative Research Network-Inflammatory Diseases Network (RiCORS-REI), Instituto de Salud Carlos III, 28029 Madrid, Spain; 12 Instituto Oftalmológico Recoletas (IOR), 41009 Seville, Spain; 13 Department of Ophthalmology, Hospital Arnau de Vilanova, 46015 Valencia, Spain; lidiaquince@hotmail.com; 14 Foundation for the Promotion of Health and Biomedical Research in the Valencian Region (FISABIO), 46020 Valencia, Spain; 15 Department of Pharmacy, Hospital of Sagunto, 46520 Sagunto, Spain; borras_joabla@gva.es; 16 Department of Ophthalmology, Consorci Sanitari Integral, 08906 Barcelona, Spain; albagomezma@gmail.com (A.G.-B.); esther.cilveti@gmail.com (E.C.); 17 Department of Ophthalmology, Cruces University Hospital, 48903 Barakaldo, Spain; soniavalserofranco@gmail.com; 18 Biobizkaia Basque Health Research Institute, 48903 Bilbao, Spain; 19 Department of Ophthalmology, Hospital Perpetuo Socorro, 06010 Badajoz, Spain; coralarriola97@gmail.com; 20 Roche Farma, 28042 Madrid, Spain; paula.garcia_lunar@roche.com (P.G.-L.); sara.kaminski@roche.com (S.K.-S.); belen.munoz_molina@roche.com (B.M.-M.); 21 Department of Ophthalmology, Clínica Universidad de Navarra, 31008 Pamplona, Spain; aglayana@unav.es; 22 Department of Ophthalmology, Universidad de Navarra, 31009 Pamplona, Spain; 23 Navarra Institute for Health Research, IdiSNA, 31008 Pamplona, Spain

**Keywords:** anti-VEGF, anti-Ang2, clinical trials, diabetic macular edema, faricimab, exudative/neovascular age-related macular degeneration, real-world

## Abstract

Neovascular age-related macular degeneration (nAMD) and diabetic macular edema (DME) are leading causes of visual impairment and require long-term intravitreal anti-vascular endothelial growth factor (VEGF) therapy. However, treatment burden and suboptimal real-world outcomes remain major challenges. Faricimab is a bispecific monoclonal antibody that simultaneously inhibits VEGF-A and angiopoietin-2 (Ang-2), a key mediator of vascular destabilization, leakage and inflammation, offering a novel dual-pathway approach aimed at improving efficacy and durability. This narrative review summarizes evidence from pivotal clinical trials and recent real-world studies evaluating the efficacy, durability, and safety of faricimab in patients with nAMD and DME. Real-world evidence from international cohorts and emerging data from Spanish routine clinical practice largely corroborate previous findings, showing anatomical improvements, stable or improved visual outcomes, and reduced treatment burden in treatment-naïve and previously treated eyes. Overall, the evidence reviewed indicates that faricimab is an effective and well-tolerated therapeutic option that may help address unmet needs in the long-term management of nAMD and DME. Importantly, data from real-world clinical practice are consistent with findings from pivotal trials, supporting the translation of faricimab’s outcomes into routine care.

## 1. Introduction

Neovascular age-related macular degeneration (nAMD) and diabetic macular edema (DME) are among the leading causes of vision impairment and blindness in the adult population [1,2]. nAMD affects mainly people over 55 years old and is responsible for blindness in nearly 2 million people and moderate-to-severe visual impairment in more than 6 million [3], while DME affects approximately 5.5% of individuals with diabetes [4].

For many years, anti-vascular endothelial growth factor (VEGF) monotherapies have represented the gold standard for the treatment of nAMD and DME [5]. Although these agents have demonstrated significant efficacy in improving visual acuity and retinal anatomy, long-term outcomes have not been consistent in both patient populations [6,7,8]. Additionally, the improvements reported in clinical trials have not been replicated in real-world practice [9], largely due to the heterogeneous patient populations in clinical settings and the lower frequency of injections typically administered compared with clinical trials [10,11].

The angiopoietin-tyrosine kinase with immunoglobulin-like and epidermal growth factor-like domains 2 (Tie2) pathway, in particular angiopoietin-2 (Ang-2), contributes to angiogenesis, vascular permeability and inflammation. Ang-2 is highly secreted under pathophysiological conditions, functioning as a negative regulator of the Ang/Tie2 axis, promoting angiogenesis, vascular destabilization and inflammation, with elevated intraocular levels of Ang-2 observed in the eyes of patients with nAMD and DME [12,13,14].

Faricimab (Vabysmo^®^, Roche, Basel, Switzerland) is a humanized intravitreal monoclonal antibody that simultaneously targets VEGF and Ang-2 pathways, without inhibiting Ang-1 [15,16]. Considering its pharmacological properties, dual-pathway inhibition and synergistic action, faricimab was developed to improve retinal outcomes and, ultimately, enhance patients’ vision [13,17]. Based on the efficacy and safety results reported in pivotal clinical trials, intravitreal faricimab received approval from the US Food and Drug Administration (FDA) and the European Medicines Agency (EMA) in 2022 for the treatment of both nAMD and DME [15,16].

In this review, we aim to summarize and discuss the most relevant evidence from pivotal clinical trials and the most recent real-world international and national studies evaluating the efficacy, durability, and safety of faricimab in patients with nAMD and DME. The purpose of incorporating Spanish real-world data alongside large international cohorts (such as FARETINA, FARWIDE, and VOYAGER) is not to compare ethnic or biological outcomes, but to illustrate how faricimab’s efficacy translates across different healthcare delivery systems. Local clinical management protocols, healthcare access, and reimbursement frameworks significantly influence real-world treatment patterns and durability. Compiling local data alongside global evidence validates the consistency and external validity of faricimab’s clinical benefits in diverse real-world settings.

## 2. Faricimab in Pivotal Clinical Trials

TENAYA and LUCERNE were two multicenter, randomized, phase 3 non-inferiority trials evaluating the efficacy and safety of faricimab versus aflibercept 2 mg in patients with nAMD. Both TENAYA and LUCERNE met their primary non-inferiority endpoints of mean change in Best-Corrected Visual Acuity (BCVA) from baseline after one year [18]. At the 2-year follow-up, BCVA gains remained similar between faricimab and aflibercept 2 mg in TENAYA (3.7 vs. 3.3 Early Treatment Diabetic Retinopathy Study [ETDRS] letters, respectively) and LUCERNE (5.0 vs. 5.2 ETDRS letters, respectively). Reductions in central subfield thickness (CST) were sustained through year 2 and remained comparable between treatment groups in both TENAYA (faricimab: −146.5 μm; aflibercept 2 mg: −146.2 μm) and LUCERNE (faricimab: −150.3 μm; aflibercept 2 mg: −141.6 μm) cohorts. These outcomes were achieved with fewer injections; the median number of injections of faricimab was 10, compared with 15 aflibercept injections. Moreover, 74.1% and 81.2% of patients from TENAYA and LUCERNE, respectively, treated with faricimab achieved extended dosing every 12 weeks (Q12W) and 16 weeks (Q16W) after two years [19]. Faricimab administered up to Q16W was well tolerated, with the incidence of ocular adverse events being similar to that observed with aflibercept 2 mg in both studies [19].

The extension study AVONELLE-X evaluated the long-term safety and tolerability of faricimab (up to 4 years) in patients with nAMD. The results of the study further support the sustained efficacy, durability and safety of faricimab; the treatment was well tolerated and no new safety signals were identified [20].

YOSEMITE and RHINE were two similarly designed multicenter, randomized, phase 3 non-inferiority clinical trials that evaluated the efficacy and safety of faricimab versus aflibercept 2 mg in patients with DME secondary to type 1 or type 2 diabetes. The primary endpoint of BCVA change was met at year 1 [21]. In year 2, BCVA gains were maintained in patients in the faricimab administered every 8 weeks (Q8W) and treat-and-extend (T&E) arms compared with those in the aflibercept 2 mg arm in both the YOSEMITE (10.7 vs. 10.7 vs. 11.4 ETDRS letters, respectively) and RHINE (10.9 vs. 10.1 vs. 9.4 ETDRS letters, respectively) trials and remained non-inferior. Regarding anatomical outcomes, reductions in CST favored faricimab over aflibercept 2 mg in both YOSEMITE (Q8W: −216.0 μm; T&E: −204.5 μm; aflibercept 2 mg: −196.3 μm) and RHINE (Q8W: −202.6 μm; T&E: −197.1 μm; aflibercept 2 mg: −185.6 μm). Similarly, a greater proportion of patients with an absence of intraretinal fluid (IRF) was observed in those treated with faricimab in YOSEMITE (Q8W: 59–63%; T&E: 43–48%; aflibercept 2 mg: 33–38%) and RHINE (Q8W: 56–62%; T&E: 45–52%; aflibercept 2 mg: 39–45%) at weeks 92–100 [22]. Faricimab was generally well tolerated, and its safety profile was comparable to that of aflibercept 2 mg [21,22].

RHONE-X, the open-label extension of the YOSEMITE/RHINE clinical trials, showed that the faricimab T&E regimen maintained vision gains, anatomic improvements and durability while being a safe therapeutic option [23].

A summary of the main efficacy and safety results from pivotal clinical trials evaluating the use of faricimab in patients with nAMD and DME is presented in Table 1.

## 3. Faricimab in Real-World Settings

### 3.1. Real-World Evidence in nAMD

Faricimab has been evaluated in multiple real-world studies that included both treatment-naïve and previously treated eyes. The two largest cohorts reported to date are the US-based FARETINA-AMD study, which included 29,035 eyes from 23,776 patients [24], and the UK-based FARWIDE-nAMD study, comprising 5582 eyes from 4667 patients [25]. Both studies included treatment-naïve and previously treated eyes [24,25].

Treatment-naïve eyes showed significant BCVA improvement from baseline in both studies (FARETINA-AMD: 57.3 ± 23.7 vs. 62.0 ± 21.3 letters at injection seven; FARWIDE-nAMD: 61 vs. 65 letters at 24 months) [24,25]. In FARETINA-AMD, treatment-naïve eyes also showed a significant reduction in CST (−53.1 ± 64.8 µm) [24]. After two years of receiving faricimab, visual acuity remained stable while CST improved from 299.0 ± 71.0 µm at baseline to 256.8 ± 48.5 µm at year 2 [26].

In previously treated eyes, BCVA remained stable from 61.7 ± 20.9 ETDRS letters at baseline to 62.0 ± 21.3 letters at injection seven in FARETINA-AMD [24] and from 69 to 68 letters in FARWIDE-nAMD [25]. Anatomically, previously treated eyes in FARETINA-AMD demonstrated a significant reduction in CST (−28.5 ± 79.7 µm) [24]. After two years of treatment, visual acuity was also stable in previously treated eyes, with improvements in CST (from 291.6 ± 86.3 µm to 259.2 ± 72.1 µm) [26].

In both studies, the number of faricimab injections declined after 6 months in treatment-naïve eyes and in previously treated eyes [24]. In FARETINA-AMD, treatment-naïve and previously treated eyes received a mean of 6.4 ± 2.6 and 7.4 ± 2.4 injections, respectively, during the first year [24]. In FARWIDE-nAMD, the number of injections in both groups decreased throughout the study with treatment-naïve and previously treated eyes receiving a median of 2 and 3 injections, respectively, in months 19–24 [25].

The ongoing prospective, industry-sponsored, VOYAGER study was designed to provide long-term follow-up for at least 5000 treatment-naïve patients [27]. Preliminary results from this study in nAMD included a total of 396 treatment-naïve and 335 previously treated eyes. After 12 months, mean visual acuity improved from baseline in treatment-naïve eyes (60.1 ± 18.5 vs. 63.7 ± 20.1 ETDRS letters), while it remained stable in previously treated eyes (66.0 ± 15.7 vs. 65.5 ± 18.1 ETDRS letters). Mean CST also improved in treatment-naïve (369.9 ± 136.0 vs. 266.0 ± 72.6 µm) and previously treated eyes (299.4 ± 99.7 vs. 291.3 ± 199.3 µm) from baseline to month 12. Investigator-reported presence of IRF and subretinal fluid (SRF) decreased 12 months after treatment in both treatment-naïve (IRF: 53.0% vs. 22.0%; SRF: 72.6% vs. 26.2%) and in previously treated eyes (IRF: 36.2% vs. 28.8%; SRF: 65.7% vs. 42.8%). The mean number of injections was 4.6 ± 1.1 in months 1–6 and 2.5 ± 1.3 in months 7–12 in treatment-naïve eyes, and 4.5 ± 1.4 and 3.3 ± 1.5, respectively, in previously treated eyes [28].

In addition to large industry-sponsored real-world evidence studies, independent real-world studies have also provided clinically relevant evidence supporting the effectiveness and safety of faricimab in routine care. The TRUCKEE study, which included a total of 376 treatment-naïve and previously treated eyes from 335 patients, showed that a single faricimab injection resulted in an improvement in BCVA and CST from baseline in treatment-naïve (BCVA: 4.9 ETDRS letters, *p* = 0.076; CST: −84.5 µm, *p* < 0.001) and in previously treated eyes (BCVA: 0.7 ETDRS letters, *p* = 0.196; CST: −25.3 µm, *p* < 0.001). After three faricimab injections, changes in BCVA and CST also improved in treatment-naïve eyes (BCVA: 8.1 ETDRS letters, *p* = 0.437; CST: −80.1 µm, *p* < 0.204) and in previously treated eyes (BCVA: 2.7 ETDRS letters, *p* = 0.045; CST: −38.1 µm, *p* < 0.001) [29]. The post hoc analysis evaluated visual and anatomic outcomes in patients switched from brolucizumab after three faricimab injections, showing that both BCVA and CST remained stable [30]. The most recent update of this study reported results on 3675 eyes across 2916 patients after four years of treatment. After nine injections, visual acuity and CST improved in previously treated eyes (BCVA: 0.75 letters, *p* = 0.94; CST: −43.68 µm, *p* < 0.00001). The prevalence of IRF and SRF in these patients was 36.8% and 39.7%, respectively [31].

Beyond the largest real-world evidence studies, independent studies from different healthcare settings have reported findings consistent with the pivotal TENAYA and LUCERNE trials, showing stable or improved visual outcomes, anatomical improvements and/or treatment-interval extension after faricimab treatment. These observations support the external validity of pivotal trial findings across diverse real-world clinical contexts [32,33,34,35,36,37].

### 3.2. Real-World Evidence in DME

As with nAMD, the largest real-world cohorts evaluating faricimab in patients with DME correspond to the FARETINA-DME and FARWIDE-DME studies. FARETINA-DME, conducted in the US, included a total of 7055 treatment-naïve and previously treated eyes from 5071 patients, with a mean follow-up of approximately 10 months [38]. FARWIDE-DME, conducted in the UK, evaluated a total of 2147 treatment-naïve and previously treated eyes from 1564 patients who received at least four faricimab injections [39].

Across both studies, changes in visual and anatomical outcomes were observed in treatment-naïve eyes and in previously treated eyes [38,39]. In FARETINA-DME, treatment-naïve eyes gained 4.3 ETDRS letters in BCVA and showed a reduction of 83.7 μm in CST after four injections [38]. Improvements in visual acuity (from 60.4 ± 19.8 to 63.1 ± 21.6 letters) were maintained after two years of treatment [40]. Consistent with these findings, a 4.7-letter gain in treatment-naïve eyes was reported in FARWIDE-DME after one year [39]. Visual acuity gains were maintained after two years (65 vs. 70 letters) [41].

Previously treated eyes experienced more modest improvements. In FARETINA-DME, these eyes gained 0.7 ETDRS letters and showed a CST reduction of 42.3 μm after four injections [38]. After two years, visual acuity changed from 65.0 ± 17.5 to 64.4 ± 20.1 letters, and mean CST decreased from 367.6 ± 117.6 μm to 301.0 ± 94.1 μm [40]. FARWIDE-DME reported a 1.2-letter improvement in this group after one year [39]. Visual acuity remained stable after two years [41].

Both studies observed that the number of faricimab injections declined after 6 months in treatment-naïve eyes and in previously treated eyes [38]. In FARETINA-DME, treatment-naïve eyes received a mean of 4.9 ± 2.7 injections during the study period, compared with 5.9 ± 2.6 in previously treated eyes [38]. Similarly, in FARWIDE-DME, treatment-naïve and previously treated eyes received a mean of 6.4 ± 1.7 and 6.9 ± 2.1 injections, respectively, with averages of 1.9 and 2.4 faricimab injections in months 7–12 [39], and a median of 1 and 2 injections, respectively, in months 19–24 [41].

The interim analysis of the VOYAGER study included 150 treatment-naïve and 109 previously treated eyes with DME. After 12 months, mean visual acuity improved from baseline in treatment-naïve eyes (61.9 ± 15.8 vs. 67.8 ± 14.3 ETDRS letters), while it remained stable in previously treated eyes (70.0 ± 11.4 vs. 69.5 ± 12.4 ETDRS letters). Mean CST also improved from baseline to month 12 in treatment-naïve (442.6 ± 145.7 vs. 271.5 ± 76.3 µm) and previously treated eyes (348.7 ± 100.9 vs. 311.4 ± 99.6 µm). Investigator-reported presence of IRF and SRF decreased 12 months after treatment in both treatment-naïve (IRF: 94.3% vs. 70.8%; SRF: 22.30% vs. 4.3%) and previously treated eyes (IRF: 80.3% vs. 66.7%; SRF: 16.0% vs. 8.6%). Treatment-naïve eyes received a mean of 4.6 ± 1.3 injections in months 1–6 and 2.3 ± 1.5 injections in months 7–12, while previously treated eyes received a mean of 4.5 and 3.3 injections in months 1–6 and 7–12, respectively [42].

The TAHOE study included 684 treatment-naïve and previously treated eyes from 442 patients. In treatment-naïve eyes, improvements were observed after three (BCVA: 9.33 ETDRS letters, *p* < 0.001; CST: −92.23 μm, *p* < 0.001) and nine injections (BCVA: 4.75 ETDRS letters, *p* = 0.21; CST: −153.68 μm, *p* < 0.001). Improvements were also observed after three (BCVA: 1.25 ETDRS letters, *p* = 0.78; CST: −24.7 μm, *p* < 0.0001) and nine injections (BCVA: 4.00 ETDRS letters, *p* = 0.03; CST: −56.69 μm, *p* < 0.001) in previously treated eyes [43].

Similarly, independent real-world studies in DME from different countries have reported functional and anatomical outcomes consistent with YOSEMITE and RHINE, further supporting the translation of faricimab efficacy, durability and safety into routine clinical practice [35,44,45,46,47,48,49].

### 3.3. Real-World Safety of Faricimab in nAMD and DME

Available safety data from real-world studies of faricimab in nAMD and DME were generally consistent with the known safety profile in the phase 3 clinical trials, without evidence of new safety signals. Reported safety outcomes primarily focused on intraocular inflammation (IOI) [24,25,26,28,29,30,31,38,39,40,41,42,43]. Overall, per injection IOI rates ranged from 0.08% to 0.14% [24,25,28,38,39,42,43], which is consistent with previous reports from larger real world studies [28]. Additionally, one case of successfully managed infectious endophthalmitis was reported [29].

The main effectiveness and safety results from real-world studies of faricimab in patients with nAMD and DME are summarized in Table 2.

### 3.4. Faricimab in Spanish Routine Clinical Practice

Considering that faricimab was approved for reimbursement in Spain in September 2023, real-world data on the effectiveness of faricimab in Spanish routine clinical practice have only recently begun to be published [50].

Overall, findings from real-world studies conducted in Spain are consistent with those reported in other settings previously described. The follow-up durations range from 4 to 24 months (with six of 12 studies evaluating outcomes between 4 and 9 months, five studies reporting 12-month outcomes, and one study reporting 24-month outcomes).

#### 3.4.1. Spanish Real-World Evidence in nAMD

Most studies evaluating the use of faricimab in patients with nAMD have primarily included patients previously treated with anti-VEGF monotherapy [51,52,53,54,55,56].

One recent study that exclusively included previously treated patients reported modest changes in BCVA. Ruiz-Medrano et al. evaluated 225 eyes from 188 patients with nAMD treated with faricimab for at least six months and reported that BCVA remained stable, with an improvement of 0.015 at the final visit. During the study period, mean treatment intervals increased from 5.9 ± 2.0 to 11.6 ± 24.3 weeks (*p* < 0.001) at the last follow-up visit. In eyes with at least a one-year follow-up (125 eyes), the mean treatment interval increased from 6.4 ± 1.9 to 11.0 ± 3.9 weeks [51]. In a 24-month prospective study by Ruiz-Medrano et al. of 110 eyes (97 patients) with pretreated nAMD, mean treatment intervals increased significantly from 6.18 ± 2.05 weeks at baseline to 13.61 ± 5.62 weeks at 2 years (*p* < 0.001), while visual acuity remained stable (0.42 ± 0.24 to 0.43 ± 0.25). At 24 months, 68.2% of eyes reached dosing intervals ≥12 weeks, 20.0% reached ≥16 weeks, and 24.6% reached ≥20 weeks [56]. An additional analysis including 232 eyes (the original 110 plus 122 eyes that were excluded because they did not meet the predefined T&E schedule) showed a significant increase in the mean treatment interval, from 7.05 ± 2.8 weeks prior to switching to 11.90 ± 5.5 weeks at the last follow-up visit (*p* < 0.001). Bernal-Morales et al. analyzed 161 previously treated eyes from 145 patients with recalcitrant nAMD who received at least one faricimab injection and were followed for a minimum of one visit. After three injections, BCVA (logarithm of the minimum angle of resolution [MAR]) remained stable. Complete resolution of IRF and SRF was observed in 18.9% and 62.2% of included eyes, respectively, after three injections. Subretinal hyperreflective material was absent in 13.5% of patients after three injections. After switching to faricimab, overall treatment intervals increased, with 37.4–38.5 of eyes reaching intervals of 8–11 weeks, 3–27% of eyes being extended to 12–15-week intervals and 0.6–9.7% of eyes being extended to ≥16 weeks [52]. Similarly, Valsero-Franco et al. evaluated faricimab effectiveness in 148 previously treated eyes with nAMD showing that visual acuity remained stable after one year. The presence of IRF decreased from 27.59% to 18.18% and subfoveal SRF from 42.53% to 17.05%. Treatment intervals improved from 77% and 7% of eyes being on ≤4 and 8–10-week intervals, respectively, to 49% of eyes reaching an 8–10-week interval after switching to faricimab (*p* < 0.001) [53].

A study by Gómez et al. evaluated 249 patients with nAMD, including 48 treatment-naïve and 221 previously treated eyes. Visual and anatomic outcomes in previously treated eyes showed modest BCVA gains (from 48 ± 13 to 49 ± 15 ETDRS letters) and CST reduction (from 296 ± 108 μm to 274 ± 110 μm) [54]. However, the analysis of the overall cohort showed greater improvements in both BCVA (from 65 ± 15 to 69 ± 14 ETDRS letters) and CST (from 316 ± 130 μm to 282 ± 59 μm) following treatment with faricimab, suggesting that treatment-naïve eyes likely experienced more pronounced improvements. Additionally, 62% of previously treated patients were able to extend their treatment intervals by four or more weeks after switching to faricimab [55]. Jiménez-Alfaro Ortego et al. analyzed 44 treatment-naïve and 59 previously treated eyes from 103 patients with nAMD. Visual acuity remained stable in both treatment-naïve and previously treated eyes. In the overall cohort, 19.4% of eyes gained ≥10 ETDRS letters after treatment with faricimab. Anatomical improvements were observed; central macular thickness (CMT) decreased in 81.6% of all eyes, with an overall median reduction of 38 μm (*p* < 0.001). The presence of IRF significantly decreased in both treatment-naïve (60.5% to 13.9%) and previously treated eyes (45.8% to 23.7%). Similarly, the presence of SRF significantly decreased in both treatment-naïve (58.1% to 13.9%) and previously treated eyes (47.5% to 18.6%) [57].

A study by Remolí-Sargues et al. included 30 eyes from 30 previously treated patients with nAMD and reported similar findings. After switching to faricimab, BCVA improved from 67.12 ± 13.77 ETDRS letters at baseline to 70.67 ± 12.53 letters at 6 months (*p* = 0.041) and 68.65 ± 13.11 letters at 12 months (*p* = 0.075). CMT also improved from 272.90 ± 87.14 μm at baseline to 215.36 ± 75.68 μm and 231.22 ± 65.93 μm at 6 and 12 months (both *p* = 0.001), respectively. Similarly, CST decreased from 196.90 ± 86.83 µm at baseline to 182.82 ± 86.85 µm and 182.22 ± 87.88 µm at 6 and 12 months, respectively, although these changes did not reach statistical significance. SRF was resolved in most patients at both 6 and 12 months (*p* = 0.002), whereas no statistically significant reduction was observed in IRF. Compared with baseline, the mean treatment interval increased by 4.93 weeks [58].

Alegre-Rueda et al. evaluated the use of faricimab in 70 eyes with nAMD previously treated with anti-VEGF therapy. After at least one injection, 53% of included eyes showed an improvement in visual acuity and a decrease in CMT [59].

The recently published study by Cornejo-Uixeda et al. reported treatment persistence and dosing intervals in 43 treatment-naïve and 105 previously treated eyes from 129 patients with nAMD. After 12 months, 48.8% of treatment-naïve and 55.5% of previously treated patients achieved 8–12-week intervals, with a mean persistence of 12.2 months (standard deviation [SD]: 0.2; 95% confidence interval [CI]: 11.8–12.6) [60].

#### 3.4.2. Spanish Real-World Evidence in DME

Esteban-Floria et al. evaluated 28 and 77 eyes from treatment-naïve and previously treated patients with DME, respectively, who received at least one faricimab injection and were followed for at least 4 months. Consistent with previous findings, the mean change in decimal BCVA from baseline to the last visit was greater in treatment-naïve eyes, with a mean gain of 0.16 compared with 0.10 in previously treated eyes. Similarly, treatment-naïve eyes experienced a greater reduction in CST (−53.7 μm) than that experienced by previously treated eyes (−37.8 μm). Overall, a mean of 5.7 ± 2.8 faricimab injections were received, with 54.5% eyes reaching a dosing interval of 8 weeks at the final visit, while 45.5% were extended to 12–16-week intervals [46].

Other studies analyzed the entire cohort of treatment-naïve and previously treated eyes with DME, showing similar outcomes. Cilveti Gómez et al. assessed faricimab effectiveness in 45 treatment-naïve and 111 previously treated eyes from 120 patients with DME. After nine months, BCVA significantly improved from 64.00 ± 12.43 to 71.1 ± 12.23 ETDRS letters, while CMT was significantly reduced from 360.99 ± 110.83 μm to 301.62 ± 109.67 μm. Extended dosing intervals of ≥Q12W were achieved in 62.8% of all eyes, and extension of treatment intervals was observed in 61.2% of previously treated patients [61]. Romero-Barranca et al. evaluated faricimab in 63 eyes from 44 patients with DME who received at least one faricimab injection and were followed for a minimum of six months. Overall, BCVA significantly improved from 53.75 ± 5.09 to 57.48 ± 5.09 ETDRS letters at the final visit. Anatomical outcomes also improved, with mean CMT decreasing from 337.71 ± 17.03 μm to 297.18 ± 17.03 μm at the final visit. Fluid biomarkers showed significant reductions: the presence of IRF decreased from 95.2% to 71.0% and hyperreflective foci (HRF) from 61.3% to 46.8% [62].

Finally, a study by Arriola included 60 treatment-naïve and 40 previously treated eyes from 85 patients of whom 40 and 20 had a history of nAMD and DME, respectively. After treatment with faricimab, decimal BCVA improved in treatment-naïve eyes (from 0.4 to 0.6 at month 4), while it remained stable in previously treated eyes (0.3 at baseline and month 4). CMT significantly decreased in both groups: from 350 μm to 150 μm in treatment-naïve eyes and from 436.5 μm to 281 μm in previously treated eyes [63]. Although in this study eyes with nAMD and DME were analyzed together, these results are in line with the other studies reported here, showing that treatment-naïve eyes experienced greater improvements compared with those previously treated.

Overall, no new safety concerns were identified across Spanish real-world studies. Reported ocular adverse events included two cases of retinal detachment [61] and two cases of uveitis that resolved after appropriate management [55,61].

A summary of the main effectiveness and safety results from studies assessing the use of faricimab in patients with nAMD and DME in Spanish real-world practice is provided in Table 3.

## 4. Discussion

The growing body of evidence from pivotal clinical trials and real-world studies supports the efficacy and effectiveness of faricimab for the treatment of both nAMD and DME. Unlike previous reviews, this narrative review places recently published and presented Spanish real-world evidence in the context of large international real-world cohorts and independent studies, providing a clinically relevant perspective on how faricimab outcomes are translating into routine practice in Spain. Importantly, this review only aims at describing faricimab under real-world conditions in Spain, rather than to compare results across different populations.

Faricimab, a bispecific antibody targeting Ang-2 and VEGF-A, has demonstrated non-inferior visual outcomes, robust anatomical improvements, and meaningful reductions in treatment burden when compared to established anti-VEGF monotherapies such as aflibercept 2 mg. Data from selected international real-world studies support the findings reported in pivotal clinical trials [32,33,34,35,36,37,44,45,46,47,48,49,64,65,66].

Data from the pivotal clinical trials TENAYA/LUCERNE (nAMD) and YOSEMITE/RHINE (DME) consistently showed that faricimab provides similar functional outcomes compared with aflibercept 2 mg, with consistent improvements in anatomical outcomes. Furthermore, in TENAYA/LUCERNE approximately 80% of faricimab-treated patients maintained 12–16-week dosing intervals after one and two years of treatment [18,19]. Similarly, the YOSEMITE/RHINE trials reported that approximately two-thirds of patients achieved dosing intervals of 12–16 weeks at one year. After two years, 78% of patients achieved 12-week dosing intervals or longer [21,22]. The extension studies AVONELLE-X (nAMD) and RHONE-X (DME) showed that faricimab was able to maintain vision and anatomic gains in the long term [20,23].

Large real-world studies investigating the effectiveness and safety of faricimab, including FARETINA, FARWIDE, and VOYAGER, demonstrated that outcomes in treatment-naïve and previously treated eyes that received faricimab were largely consistent with those from clinical trials. Similarly, studies conducted in routine clinical practice in Spain further corroborate these findings [51,67,68,69,70,71,72]. Overall, real-world studies showed improvements in visual acuity, retinal thickness and retinal fluid, even in more heterogeneous patient populations. Importantly, real-world evidence confirms that faricimab’s durability and anatomical benefits translate into routine clinical practice. In this context, it is important to note that not all earlier-generation anti-VEGF monotherapies have been able to reproduce, in real-world settings, the outcomes reported in clinical trials. Several anti-VEGF agents have demonstrated lower visual acuity gains in routine clinical practice. This discrepancy is likely attributable to the reduced monitoring frequency and lower number of injections that patients typically receive in real-world practice, which ultimately results in suboptimal visual outcomes [10,73,74,75].

Available real-world evidence on faricimab treatment for patients with nAMD and DME shows that patients who transition to faricimab after suboptimal response to other anti-VEGF agents can achieve improved anatomical outcomes, including fluid resolution, stabilization or gains in visual acuity, and longer treatment intervals compared with prior therapy. These findings support the idea that VEGF signaling is not the only altered pathway in eyes with nAMD and DME and align with expert recommendations suggesting that faricimab’s dual inhibition may provide additional benefit in patients insufficiently controlled with VEGF inhibitors alone [76]. Consistently, reviewed real-world data indicate that while both groups benefit, treatment-naïve eyes experienced more pronounced gains in visual acuity and anatomical outcomes compared with previously treated eyes. In addition, a recent publication has reported that resolution of subretinal hyperreflective material was observed in 70% of treatment-naïve eyes with nAMD, which was higher than that reported for aflibercept (31.4% to 47.6%), further supporting the potential added effect of Ang-2 inhibition [77].

These observations are further supported by biomarker analyses from pivotal phase 3 trials. Beyond visual acuity outcomes, post hoc analyses from four phase 3 trials in nAMD and DME demonstrate that dual Ang-2/VEGF-A inhibition with faricimab may provide greater control of vascular instability biomarkers than aflibercept 2 mg. This is evidenced by an enhanced capacity to control neovascularization and vascular leakage (leading to greater resolution of retinal fluid, pigment epithelial detachment, and macular leakage) while simultaneously targeting inflammation (through HRF reduction in DME), and fibrotic proliferation (by reducing the risk of epiretinal membrane [ERM] development in DME). Rather than being solely a consequence of faricimab’s higher molar anti-VEGF binding capacity, these biomarker improvements are hypothesized to be driven by Ang-2 inhibition. Ultimately, these anatomical gains translate into superior disease control and extended treatment durability [13].

Overall, the improvements reported in both phase 3 clinical trials and real-world studies in treatment-naïve eyes receiving faricimab suggest that inhibition of VEGF and Ang-2 pathways may lead to better visual and anatomical outcomes compared with initiating therapy in patients who have already been treated with other therapeutic options.

One of faricimab’s clinical advantages is its capacity to extend dosing intervals up to 16 weeks, demonstrated across both phase 3 clinical trials and real-world cohorts. In this regard, a recently published systematic review and meta-analysis of intravitreal therapies for nAMD reported that faricimab is a therapeutic option that provides an optimal balance between improvement in visual and anatomic outcomes and the number of injections or visits required to manage the disease [78]. Thus, this extended durability may reduce patient burden and improve long-term adherence, a critical challenge in chronic retinal diseases. In this context, a recent Spanish study reported that, beyond extending treatment intervals, switching patients to faricimab reduced the number of annual visits, resulting in economic savings, mainly associated with a reduced clinical workload [79]. Consistent with these observations, a recent Spanish real-world pharmacoeconomic study showed that switching patients with nAMD to faricimab generated total direct healthcare cost savings of €633,226.06 in a cohort of 225 eyes treated under a treat-and-extend regimen with baseline treatment intervals of less than 12 weeks [51,80].

Faricimab has demonstrated a favorable and consistent safety profile across pivotal phase 3 clinical trials in the treatment of both nAMD and DME, with an incidence of ocular and systemic adverse events comparable to those observed with aflibercept 2 mg. Emerging real-world evidence from international and national cohorts included in this review similarly reported that faricimab was well tolerated, corroborating the safety findings observed in controlled trials. Importantly, a recent real-world safety meta-analysis including more than 1.4 million faricimab injections showed rates of IOI and endophthalmitis consistent with those observed in the VOYAGER study [28]. No new safety issues have been identified beyond those reported in clinical trials or in other real-world studies [81,82,83].

Despite encouraging findings, this narrative review has limitations. The real-world studies discussed here were selected to prioritize large international cohorts, independent studies with clinically relevant findings, and available Spanish routine-practice evidence; therefore, this review should not be interpreted as a systematic review of all published faricimab real-world studies. Unlike clinical trials, real-world studies typically include heterogeneous populations, which, although more representative of routine clinical practice, may influence study outcomes, particularly in studies with small sample sizes. Additionally, the effect of ethnic variations must be acknowledged when extrapolating the results of such trials and global studies. For instance, polypoidal choroidal vasculopathy (PCV), a subtype of nAMD with distinct treatment responses, is significantly more prevalent in Asian cohorts than in Caucasian populations. Although subgroup analyses from pivotal trials (such as the Japan subgroup in TENAYA) and Asian real-world studies demonstrate consistent efficacy, caution is warranted when generalizing outcomes across ethnically diverse groups. Furthermore, current clinical trials and real-world studies are inherently constrained by reliance on functional (BCVA) and basic anatomical (CST) primary endpoints. Emerging evidence emphasizes that CST alone does not fully reflect disease activity or structural integrity. Novel optical coherence tomography-based biomarkers, including intraretinal and subretinal fluid (IRF, SRF) volume, subretinal hyperreflective material, HRF, pigment epithelial detachment (PED) volume, and choroidal vascularity index, provide more granular insights into vascular stability, inflammation, and fibrotic risk. Future studies integrating these advanced biomarkers will be essential to better evaluate functional and structural outcomes under dual Ang-2/VEGF-A inhibition. While large international datasets now provide follow-up of up to 2 years, the Spanish evidence remains more limited; most Spanish real-world studies included in this review had variable and relatively short follow-up durations (60% of studies reporting outcomes at ≤9 months and only a few reaching 12 months), limiting the comparability of results and the ability to assess long-term effectiveness and safety. However, newly published prospective data now extend local follow-up to 24 months. In this 2-year Spanish study, treatment-interval extensions were sustained through 104 weeks, reinforcing that short-term anatomical gains translate into long-term durability in local clinical practice. Furthermore, several studies were conducted at single centers, limiting the generalizability of their findings. Some datasets may represent interim analyses with incomplete datasets, and therefore conclusions must be interpreted with caution. Additional Spanish real-world studies with larger cohorts and longer follow-up periods will be essential to strengthen the evidence base for faricimab in nAMD and DME and to further confirm its clinical benefits and safety profile in the local setting.

## 5. Conclusions

Faricimab has demonstrated visual and anatomical improvements in nAMD and DME while enabling extended treatment intervals that may reduce patient burden in clinical trials and in real-world settings. Moreover, the real-world safety profile is consistent with the established safety profile reported in pivotal trials, highlighting a favorable benefit–risk profile. Overall, real-world effectiveness outcomes with faricimab appear to be consistent with those observed in pivotal clinical trials, supporting the translation of trial findings into routine clinical practice.

## Figures and Tables

**Table 1:** Summary of phase III clinical trials of faricimab in nAMD and DME.

Study	Design	Pathology	Patients	Key Findings at 1 Year	Key Findings at 2 Years
TENAYA [18,19]	Phase 3, randomized, non-inferiority	nAMD	334 faricimab up to Q16W 337 aflibercept Q8W	Adjusted mean BCVA change (95% CI) 5.8 ETDRS letters (4.6 to 7.1) faricimab, 5.1 ETDRS letters (3.9 to 6.4) aflibercept. Adjusted mean CST change (95% CI) −136.8 µm (−142.6 to −131.0) faricimab, −129.4 µm (−135.2 to −123.5) aflibercept. Incidence of ocular events 36.3% faricimab, 38.1% aflibercept.	Adjusted mean BCVA change (95% CI) 3.7 ETDRS letters (2.1 to 5.4) faricimab, 3.3 ETDRS letters (1.7 to 4.9) aflibercept. Adjusted mean CST change (95% CI) −146.5 µm (−152.7 to −140.3) faricimab, −146.2 µm (−152.4 to −140.1) aflibercept. Incidence of ocular events 55.0% faricimab, 56.5% aflibercept.
LUCERNE [18,19]	Phase 3, randomized, non-inferiority	nAMD	331 faricimab up to Q16W 327 aflibercept Q8W	Adjusted mean BCVA change (95% CI) 6.6 ETDRS letters (5.3 to 7.8) faricimab, 6.6 ETDRS letters (5.3 to 7.8) aflibercept. Adjusted mean CST change (95% CI) −137.1 µm (−143.1 to −131.2) faricimab −130.8 µm (−136.8 to −124.8) aflibercept. Incidence of ocular events 40.2% faricimab, 36.2% aflibercept.	Adjusted mean BCVA change (95% CI) 5.0 ETDRS letters (3.4 to 6.6) faricimab, 5.2 ETDRS letters (3.6 to 6.8) aflibercept. Adjusted mean CST change (95% CI) −150.3 µm (−156.1 to −144.6) faricimab, −141.6 µm (−147.5 to −135.7) aflibercept. Incidence of ocular events 52.9% faricimab, 47.5% aflibercept.
YOSEMITE [21,22]	Phase 3, randomized, non-inferiority	DME	315 faricimab Q8W 313 faricimab PTI 312 aflibercept Q8W	Adjusted mean BCVA change (97.52% CI) 10.7 ETDRS letters (9.4 to 12.0) faricimab Q8W, 11.6 ETDRS letters (10.3 to 12.9) faricimab PTI, 10.9 ETDRS letters (9.6 to 12.2) aflibercept Q8W. Adjusted mean CST change (95.04% CI) −206.6 μm (−214.7 to −198.4) faricimab Q8W, −196.5 μm (−204.7 to −188.4) faricimab PTI, −170.3 μm (−178.5 to −162.2) aflibercept Q8W. Absence of IRF 42–48% faricimab Q8W, 34–43% faricimab PTI, 22–25% aflibercept Q8W. Incidence of ocular events 31% faricimab Q8W, 34% faricimab PTI, 33% aflibercept Q8W.	Adjusted mean BCVA change (95.04% CI) 10.7 ETDRS letters (9.4 to 12.1) faricimab Q8W, 10.7 ETDRS letters (9.4 to 12.1) faricimab T&E *, 11.4 ETDRS letters (10.0 to 12.7) aflibercept Q8W (*p* > 0.05). Adjusted mean CST change (95.04% CI) −216.0 μm (−224.0 to −208.0) faricimab Q8W, −196.3 μm (−204.3 to −188.2) aflibercept Q8W (*p* = 0.0007). In the faricimab T&E arm *, CST change (−204.5 μm [−212.4 to −196.5]) was comparable with aflibercept (*p* > 0.05). Absence of IRF 59–63% faricimab Q8W, 43–48% faricimab T&E *, 33–38% aflibercept Q8W. Incidence of ocular events 47% faricimab Q8W, 47% faricimab T&E *, 46% aflibercept Q8W.
RHINE [21,22]	Phase 3, randomized, non-inferiority	DME	317 faricimab Q8W 319 faricimab PTI 315 aflibercept Q8W	Adjusted mean BCVA change (97.52% CI) 11.8 ETDRS letters (10.6 to 13.0) faricimab Q8W, 10.8 ETDRS letters (9.6 to 11.9) faricimab PTI, 10.3 ETDRS letters (9.1 to 11.4) aflibercept Q8W. Adjusted mean CST change (95.04% CI) −195.8 μm (−204.1 to −187.5) faricimab Q8W, −187.6 μm (−195.8 to −179.5) faricimab PTI, −170.1 μm (−178.3 to −161.8) aflibercept Q8W. Absence of IRF 39–43% faricimab Q8W, 33–41% faricimab PTI, 23–29% aflibercept Q8W. Incidence of ocular events 43% faricimab Q8W, 37% faricimab PTI, 36% aflibercept Q8W.	Adjusted mean BCVA change (95.04% CI) 10.9 ETDRS letters (9.5 to 12.3) faricimab Q8W, 10.1 ETDRS letters (8.7 to 11.5) faricimab T&E *, 9.4 ETDRS letters (7.9 to 10.8) aflibercept Q8W (*p* > 0.05). Adjusted mean CST change (95.04% CI) −202.6 μm (−211.1 to −194.2) faricimab Q8W, −185.6 μm (−194.1 to −177.1) aflibercept Q8W (*p* = 0.0052). In the faricimab T&E arm *, CST change (−197.1 μm [−205.3 to −188.9]) was comparable with aflibercept (*p* > 0.05). Absence of IRF 56–62% faricimab Q8W, 45–52% faricimab T&E *, 39–45% aflibercept Q8W. Incidence of ocular events 52% faricimab Q8W, 52% faricimab T&E *, 45% aflibercept Q8W.

BCVA: best-corrected visual acuity; CI: confidence interval; CST: central subfield thickness; DME: diabetic macular edema; ETDRS: early treatment diabetic retinopathy study; IRF: intraretinal fluid; nAMD: neovascular age-related macular degeneration; PTI: personalized treatment intervals; Q8W: every 8 weeks; Q16W: every 16 weeks; T&E: treat and extend. * PTI (Personalized Treatment Intervals) represents the treat-and-extend (T&E) dosing regimen evaluated in YOSEMITE and RHINE.

**Table 2:** Summary of international real-world studies of faricimab in nAMD and DME.

Study	Design	Pathology	Number of Eyes (Patients)	Key Findings
FARETINA-AMD [24]	Retrospective, chart review	nAMD	29,035 (23,776)	In treatment-naïve eyes, the mean (SD) BCVA significantly improved from 57.3 (23.7) ETDRS letters at baseline to 62.0 (21.3) ETDRS letters after faricimab injection seven (*p* < 0.001). The mean (SD) CST was significantly reduced from 307.6 (77.8) μm at baseline to 253.7 (54.0) μm at injection seven (*p* < 0.0001). In previously treated eyes, the mean (SD) BCVA remained stable from baseline (61.7 [20.9] ETDRS letters) to injection seven (62.0 [23.2] ETDRS letters) (*p* > 0.05). The mean (SD) CST was significantly reduced from 293.1 (94.2) μm at baseline to 270.7 (82.7) μm at injection seven (*p* < 0.0001). IOI/injection for treatment-naïve and previously treated eyes was 0.13% and 0.11%, respectively.
FARWIDE-nAMD [25]	Multicentric, retrospective, observational	nAMD	5582 (4667)	In treatment-naïve eyes, median (IQR) BCVA improved from 61 (50 to 70) ETDRS letters at baseline to 65 (46 to 74) ETDRS letters at 24 months. In previously treated eyes, median (IQR) BCVA changed from 69 (57 to 76) ETDRS letters at baseline to 68 (54 to 76) ETDRS letters at 24 months.
TRUCKEE [30]	Retrospective, chart review	nAMD	56 (49)	The mean (SD) BCVA remained stable from 58.8 (18.2) ETDRS letters at baseline to 60.9 (16.1) after one injection (*p* = 0.191). The mean (SD) BCVA remained stable from 59.0 (17.7) ETDRS letters at baseline to 60.6 (16.1) after three injections (*p* = 0.174). The mean (SD) CST was significantly reduced from 317.4 (105.1) μm at baseline to 312.2 (107.1) μm after one injection (*p* = 0.048). The mean (SD) CST remained stable from 309.1 (102.8) μm at baseline to 297.8 (94.0) μm after three injections (*p* = 0.086).
VOYAGER-nAMD [28]	Multicentric, prospective, observational	nAMD	731	In treatment-naïve eyes, mean VA (SD) changed from 60.1 (18.5) ETDRS letters at baseline to 63.7 (20.1) letters at month 12. Mean CST (SD) changed from 369.9 (136.0) µm at baseline to 266.0 (72.6) µm at month 12. The prevalence of SRF decreased from 72.6% at baseline to 26.2% after 12 months. The prevalence of IRF decreased from 53.0% at baseline to 22.0% after 12 months. In previously treated eyes, mean VA (SD) changed from 66.0 (15.7) ETDRS letters at baseline to 65.5 (18.1) letters at month 12. Mean CST (SD) changed from 299.4 (99.7) µm at baseline to 291.3 (199.3) µm at month 12. The prevalence of SRF decreased from 65.7% at baseline to 42.8% after 12 months. The prevalence of IRF decreased from 36.2% at baseline to 28.8% after 12 months. IOI/injection was 0.13%.
Cattaneo [32]	Single-center, retrospective study	nAMD	65 (65)	BCVA improved at the 12-month follow-up in naïve eyes (+6.9 ETDRS letters from baseline, *p* = 0.053) and was maintained in switch eyes. Significant reductions in SRF and IRF volumes were noted in both groups. A significant reduction in PED volume was observed over the follow-up period in both groups (mean slope = −206 nL, 95%CL = −273/−138; *p* < 0.001). No cases of intraocular inflammation were observed.
Kunzmann [33]	Single-center, observational, retrospective study	nAMD	170 (136)	After one IVI of faricimab, previously treated (n = 160) eyes and treatment-naïve (n = 10) eyes showed a mean BCVA gain of +0.59 ± 0.52 letters (*p* = 0.364) and +5.00 ± 6.50 letters (*p* = 0.461), and a mean CST reduction of −27.65 ± 5.33 µm (*p* < 0.001) and −94.10 ± 39.74 μm (*p* = 0.042), respectively. After three IVIs of faricimab, previously treated (n = 106) and treatment-naïve (n = 5) eyes showed a mean BCVA increase of +1.57 ± 0.88 letters (*p* = 0.051) vs. +12.50 ± 8.14 letters (*p* = 0.185), and a mean CST reduction of −25.51 ± 5.82 µm (*p* < 0.001) vs. −82.60 ± 36.20 µm from baseline, respectively. Mean PED was also significantly reduced even between the first and the third faricimab injection in previously treated eyes (*p* = 0.03). The proportion of eyes with IRF and SRF improved significantly in all eyes. Ocular AEs were reported in three out of 170 eyes, and one patient had two stroke events during faricimab therapy.
Sattah [34]	Single-center, retrospective study	nAMD	84 (64)	Following faricimab initiation, eyes had a reduced mean ± SE CMT (282.3 ± 16.2 μm vs. 244.8 ± 14.3 μm; *p* < 0.01). Annual injection frequency increased from 7.73 ± 0.33 to 8.66 ± 0.28 injections (*p* < 0.001). VA did not change significantly during the year before faricimab initiation (*p* = 0.539) but decreased after initiation (from 0.56 ± 0.05 logMAR to 0.66 ± 0.06 logMAR; *p* < 0.01). Four eyes developed macular atrophy following faricimab initiation (*p* < 0.01).
Kniggendorf [35]	Multicenter, retrospective chart review	nAMD/DME	220 (181)	After three injections, the mean BCVA improved to 61.06 for nAMD (*p* < 0.001; average gain of 3.97 letters) and 70.27 for DME (*p* < 0.001; average gain of 6.73 ETDRS letters). The baseline mean CST was 320.4 μm (nAMD) and 380.4 μm (DME), decreased to 264.3 μm (nAMD; *p* < 0.001; − 56.1 μm) and 312.7 μm (DME; *p* < 0.001; − 67.7 μm). Differences in BCVA and CST remained statistically significant when subgroups of naïve or previously treated eyes were analyzed. All reported AEs occurred in nAMD patients and included submacular hemorrhage (2 eyes), RPE tear (1 eye) and increased intraocular pressure (1 eye). No intraocular inflammation was reported.
Tanaka [36]	Single-center, prospective, non-randomized, observational study	nAMD	24	Significant BCVA (*p* = 4.9 × 10^−4^) and CRT (*p* = 1.3 × 10^−5^) improvements were observed post-faricimab treatment. Faricimab achieved dry macula in 77.3% of eyes, with SRF resolution in most cases, although IRF often persisted.
Agrawal [37]	Single-center, retrospective observational study	nAMD/DME	42 (42)	Faricimab significantly improved BCVA and reduced CMT across all groups after a mean follow-up period of 33.31 (±7.41) weeks. DME patients’ BCVA improved from 0.71 (±0.36) logMAR to 0.46 (±0.35) logMAR (*p* < 0.0001), nAMD from 1.24 (±0.73) to 0.43 (±0.43) logMAR (*p* = 0.00003). CMT decreased from 454.43 (±164.76) µm to 255.3 (±81.17) µm (*p* < 0.00001) overall. Significant reductions were also observed in IRF and SRF in nAMD patients, with IRF decreasing from 48% to 16% (*p* = 0.008) and SRF from 100% to 20% (*p* < 0.00001). No significant AEs, including intraocular inflammation (IOI), were reported.
FARETINA-DME [38]	Retrospective, chart review	DME	7055 (5205)	In treatment-naïve eyes, the mean (SD) BCVA significantly improved from 60.3 (19.8) ETDRS letters at baseline to 63.9 (18.5) ETDRS letters after faricimab injection four (*p* < 0.01). The mean (SD) CST was significantly reduced from 359.6 (115.0) μm at baseline to 307.4 (114.6) μm at injection four (*p* < 0.01). In previously treated eyes, the mean (SD) BCVA was significantly increased from 64.0 (18.3) ETDRS letters at baseline to 65.3 (19.0) ETDRS letters at injection four (*p* < 0.01). The mean (SD) CST was significantly reduced from 364.3 (132.2) μm at baseline to 330.1 (121.2) μm at injection four (*p* < 0.01). IOI/injection for previously treated and naïve eyes was 0.11% and 0.14%, respectively.
FARWIDE-DMO [39]	Multicentric, retrospective, observational	DME	2147 (1564)	Mean VA change (95% CI) from baseline to 12 months was 4.7 (3.4 to 5.9) letters for naïve eyes (*p* < 0.001) and 1.2 (0.6 to 1.9) letters for previously treated eyes (*p* < 0.001). 81 intraocular inflammation events in naïve (0.14% IOI/injection) and 137 IOI events in previously treated eyes (0.14% IOI/injection).
VOYAGER-DME [42]	Multicentric, prospective, observational	DME	259 (194)	In treatment-naïve eyes, the mean VA (SD) changed from 61.9 (15.8) ETDRS letters at baseline to 67.8 (14.3) letters at month 12. Mean CST (SD) changed from 442.6 (145.7) µm at baseline to 271.5 (76.3) µm at month 12. The prevalence of SRF decreased from 22.3% at baseline to 4.3% after 12 months. The prevalence of IRF decreased from 94.3% at baseline to 70.8% after 12 months. In previously treated eyes, mean VA (SD) changed from 70.0 (11.4) ETDRS letters at baseline to 69.5 (12.4) letters at month 12. Mean CST (SD) changed from 348.7 (100.9) µm at baseline to 311.4 (99.6) µm at month 12. The prevalence of SRF decreased from 16.0% at baseline to 8.6% after 12 months. The prevalence of IRF decreased from 80.3% at baseline to 66.7% after 12 months. IOI/injection was 0.09%.
TAHOE [43]	Multi-center, retrospective study	DME	684 (442)	In treatment-naïve eyes, improvements were observed after three (BCVA: 9.33 ETDRS letters, *p* < 0.001; CST: −92.23 μm, *p* < 0.001) and nine injections (BCVA: 4.75 ETDRS letters, *p* = 0.21; CST: −153.68 μm, *p* < 0.001). In previously treated eyes improvements were also observed after three (BCVA: 1.25 ETDRS letters, *p* = 0.78; CST: −24.7 μm, *p* < 0.0001) and nine injections (BCVA: 4.00 ETDRS letters, *p* = 0.03; CST: −56.69 μm, *p* < 0.001) in previously treated eyes
Mishra [44]	Randomized controlled trial conducted at a tertiary care institute	DME	74 (74)	At 48 weeks, mean BCVA improvement was similar (aflibercept: −0.366 logMAR; faricimab: −0.374 logMAR, *p* = non-significant) between the aflibercept 2 mg and the faricimab treatment groups. CMT reduction was equivalent between groups (aflibercept: −119 µm; faricimab: −132 µm, *p* = non-significant). Faricimab achieved significantly longer mean dosing intervals (12.86 vs. 9.47 weeks, *p* < 0.001) with fewer injections required during the maintenance phase. Safety profiles were similar, with only mild, transient AEs reported.
Al-Rufayie [45]	Single-center, retrospective study	DME	76 (60)	Treatment-naïve patients showed significant improvement in BCVA from 0.86 ± 0.31 to 0.23 ± 0.26 logarithm of the minimum angle of resolution (logMAR) and a reduction in CST from 385.71 ± 103.59 to 299.14 ± 76.00 μm. Previously treated patients also demonstrated improvements, with BCVA enhancing from 0.43 ± 0.38 to 0.31 ± 0.34 logMAR and CST decreasing from 427.00 ± 129.40 to 318.88 ± 89.40 μm. Few AEs were noted, affirming the safety profile of faricimab.
Chakraborty [47]	Multicenter observational study	DME	39 (39)	Significant improvements in BCVA were observed in both treatment-naïve and recalcitrant groups, with greater gains in the naïve group (*p* < 0.001). Overall, BCVA improved from 0.48 logMAR to 0.27 logMAR (*p* < 0.001), and 59% of eyes gained more than 10 ETDRS letters and 41% gained > 15 ETDRS letters. Both groups showed a significant reduction in CMT, with the naïve group achieving greater reduction (*p* < 0.001). The overall CMT reduction was statistically significant at 6 months (*p* < 0.001). Resolution of IRF and SRF was achieved in both groups, with SRF reducing from 82.1% to 20.5% (*p* < 0.001) and IRF from 87.2% to 17.9% (*p* < 0.001). Significant reductions in HRF were also observed across both inner (*p* < 0.001) and outer retinal layers (*p* < 0.001). No ocular or systemic AEs were reported.
Huber [48]	Single-center retrospective study	DME	52 (40)	After 12 weeks of the switch from aflibercept 2 mg to faricimab, 54% of rDME eyes and 25% of nrDME eyes showed a CST decrease of >20% or CST ≤ 250 µm. 73% of rDME eyes and 47% of nrDME eyes reached an extended interval of 8 weeks or longer after 12 weeks.
Saima [49]	Single-center retrospective study	DME	15 (15)	The change in VA showed no significant difference after switching from aflibercept 2 mg to faricimab (*p* = 0.732). The mean CMT decreased significantly during the follow-up period (both *p* < 0.001). MBR showed no significant difference during the follow-up period (*p* = 0.26). However, it decreased significantly 4 weeks after faricimab (*p* = 0.01) compared with the baseline value, but not 4 weeks after aflibercept 2 mg (*p* = 0.074). A significant association was observed between decreased MBR and decreased CMT in patients who received faricimab (correlation coefficient: 0.501, *p* = 0.005) but not in those who received aflibercept 2 mg (*p* = 0.735).

AEs: adverse events; BCVA: best-corrected visual acuity; CI: confidence interval; CRT: central retinal thickness; CST: central subfield thickness; DME: diabetic macular edema; ETDRS: early treatment diabetic retinopathy study; HRF: hyperreflective foci; IOI: intraocular inflammation; IQR: interquartile range; IRF: intraretinal fluid; IVI: intravitreal injection; MAR: minimum angle of resolution; MBR: mean blur rate; nAMD: neovascular age-related macular degeneration; nrDME: non-responding diabetic macular edema; rDME: responding diabetic macular edema; PED: pigment epithelial detachment; SD: standard deviation; SRF: subretinal fluid; VA: visual acuity. HRF (hyperreflective foci) is believed to be associated with retinal inflammation and may predict a poor visual prognosis in DME [47]; PED (pigment epithelial detachment) is a clinically visible separation of the RPE monolayer from the underlying Bruch’s membrane [32]; MBR (mean blur rate) is a measure of relative blood flow velocity in the optic nerve papilla and retinal vessels derived from contrast pattern variations [49]; nrDME (non-responding diabetic macular edema) represents DME with persistent fluid despite anti-VEGF therapy, indicating a need for dual Ang-2/VEGF inhibition [47].

**Table 3:** Summary of Spanish real-world studies of faricimab in nAMD and DME.

Study	Design	Pathology	Number of Eyes (Patients)	Follow-Up Duration	Key Findings
Ruiz-Medrano [51]	Prospective, observational	nAMD	225 (188)	Mean (SD) follow-up of 51.4 (11.8) weeks	The mean (SD) BCVA remained stable from 0.41 (0.59) at baseline to 0.43 (0.58) at the last follow-up visit (*p* = 0.0112). The mean (SD) treatment intervals increased from 5.9 (2.0) to 11.6 (24.3) weeks at last follow-up visit (*p* < 0.001). No reported serious AEs.
Ruiz-Medrano [56]	Prospective, observational	nAMD	110 (97)	24 months	The mean (SD) treatment intervals increased from 6.18 (2.05) at baseline to 13.61 (5.62) weeks at 2 years (*p* < 0.001). Visual acuity remained stable: from 0.42 (0.24) to 0.43 (0.25). At 24 months, 68.2% of eyes reached dosing intervals ≥ 12 weeks, 20.0% reached ≥16 weeks, and 24.6% reached ≥20 weeks.
Bernal-Morales [52]	Single-center, retrospective, observational	nAMD	161 (145)	Mean (SD) follow-up of 8.3 (2.5) months	The mean (SD) BCVA (logMAR) remained stable after three injections (−0.10 [0.41], *p* = 0.132). IRF resolution occurred in 18.9% of eyes after three injections (*p* = 0.008). SRF resolution occurred in 62.2% of eyes after three injections (*p* < 0.001). No reported AEs.
Valsero-Franco [53]	Multicentric, retrospective, observational	nAMD	148	12 months	The median (IQR) VA remained stable from 0.5 (0.3–0.7) at baseline to 0.5 (0.3–0.7) after treatment with faricimab. IRF prevalence decreased from 27.59% at baseline to 18.18% after treatment with faricimab. SRF prevalence decreased from 42.53% at baseline to 17.05% after treatment with faricimab.
Gómez [54,55]	Single-center, retrospective, observational	nAMD	269 (249)	At least 9 months	The overall mean (SD) BCVA remained stable from 65 (15) ETDRS letters at baseline to 69 (14) ETDRS letters after treatment with faricimab (*p* = 0.08). In previously treated eyes, the mean (SD) BCVA remained stable from baseline (48 [13] ETDRS letters) to the final visit (49 [15] ETDRS letters) (*p* = 0.31). The overall mean (SD) CST was significantly reduced from 316 (130) μm at baseline to 282 (59) μm after treatment with faricimab (*p* = 0.03). In previously treated eyes, the mean (SD) CST remained stable from baseline (296 [108] μm) to the final visit (274 [110] μm) (*p* = 0.17). Overall, IRF and/or SRF prevalence decreased from 77.93% at baseline to 24.38% after treatment with faricimab. In previously treated eyes, IRF prevalence significantly decreased from 43% at baseline to 26% at the final visit (*p* = 0.002). SRF prevalence significantly decreased from 63% at baseline to 30% at the final visit (*p* < 0.001).
Jiménez-Alfaro Ortego [57]		nAMD	103 (103)	Mean (SD) follow-up of 13.6 (4.7) weeks	In treatment-naïve eyes, median (IQR) BCVA remained stable from 61 (35–71.5) ETDRS letters at baseline to 61.5 (42.5–76) letters after treatment with faricimab; median (IQR) BCVA (logMAR) remained stable from 0.40 (0.19–0.82) to 0.35 (0.13–0.91). The median CMT decreased by −49 μm after treatment with faricimab. IRF prevalence decreased from 60.5% at baseline to 13.9% after treatment with faricimab (*p* < 0.001). SRF prevalence decreased from 58.1% at baseline to 13.9% after treatment with faricimab (*p* < 0.001). In previously treated eyes, median (IQR) BCVA remained stable from 68 (55–74) ETDRS letters at baseline to 68 (49–75) letters after treatment with faricimab; median (IQR) BCVA (logMAR) remained stable from 0.30 (0.15–0.70) to 0.30 (0.15–0.52). The median CMT decreased by −33 μm after treatment with faricimab. IRF prevalence decreased from 45.8% at baseline to 23.7% after treatment with faricimab (*p* = 0.01). SRF prevalence decreased from 47.5% at baseline to 18.6% after treatment with faricimab (*p* < 0.001).
Remolí-Sargues [58]	Single-center, retrospective, observational	nAMD	30 (30)	Up to 12 months	The mean (SD) BCVA improved from 67.12 (13.77) ETDRS letters at baseline to 70.67 (12.53) at 6 months (*p* = 0.041) and remained stable at 12 months (68.65 [13.11] letters, *p* = 0.075). The mean CMT decreased from 272.90 (87.14) μm at baseline to 215.36 (75.68) μm at 6 months and 231.22 (65.93) μm at 12 months (both *p* = 0.001). The mean CST decreased from 196.90 (86.83) μm at baseline to 182.82 (86.85) μm at 6 months (*p* = 0.080) and 182.22 (87.88) μm at 12 months (*p* = 0.055). SRF resolved in most patients at both 6 and 12 months (*p* = 0.002). IRF prevalence decreased from 30.0% at baseline to 16.7% and 20.0% at 6 and 12 months, respectively (*p* = 0.135). No reported AEs.
Alegre-Rueda [59]	Single-center, retrospective, observational	nAMD	70 (70)	At least 3 months	The majority of eyes showed an improvement in VA (53%), 29% remained the same and only 11% worsened. 53% of eyes showed a decrease in CMT. One case of endophthalmitis was observed after two faricimab injections, which was treated with intravitreal antibiotics and resolved.
Cornejo-Uixeda [60]	Single-center, retrospective, observational	nAMD	148 (129)	Up to 12 months	48.8% of treatment-naïve and 55.5% of pretreated patients achieved 8–12-week intervals. Mean persistence was 12.2 months (SD 0.2; 95% CI: 11.8–12.6). Persistence rate was 93% at 6 and 12 months. No serious AEs or endophthalmitis occurred.
Esteban-Floria [46]	Single-center, retrospective, observational	DME	105 (79)	Mean (SD) follow-up of 10.0 (3.1) months	In treatment-naïve eyes, the mean (SD) BCVA (decimal) significantly improved from 0.59 (0.11) at baseline to 0.75 (0.29) at the final visit (*p* = 0.003). The mean (SD) CFT was significantly reduced from 350.1 (87.9) μm at baseline to 296.4 (64.3) μm at the final visit (*p* < 0.001). In previously treated eyes, the mean (SD) BCVA (decimal) significantly improved from 0.46 (0.21) at baseline to 0.56 (0.24) at the final visit (*p* = 0.026). The mean (SD) CFT was significantly reduced from 328.0 (87.2) μm at baseline to 290.2 (61.3) μm at the final visit (*p* = 0.001). No ocular AEs.
Cilveti-Gómez [61]	Single-center, retrospective, observational	DME	156 (120)	9 months	The overall mean (SD) BCVA significantly improved from 64.00 (12.43) ETDRS letters at baseline to 71.14 (12.23) ETDRS letters at 9 months (*p* = 0.029). The overall mean (SD) CMT was significantly reduced from 360.99 (110.83) μm at baseline to 301.62 (109.67) μm at 9 months (*p* = 0.041). Two retinal detachments and one uveitis were reported.
Romero-Barranca [62]	Single-center, retrospective, observational	DME	63 (44)	At least 6 months	Mean (SD) ETDRS scores improved significantly from 53.75 (5.09) letters at baseline to 57.48 (5.09) letters at the final visit (*p* = 0.023). The mean (SD) CMT decreased from 337.71 (17.03) μm at baseline to 297.18 (17.03) μm at the final visit (*p* < 0.001). IRF prevalence decreased from 95.2% at baseline to 71.0% at the final visit (*p* < 0.001). HRF prevalence decreased from 61.3% at baseline to 46.8% at the final visit (*p* < 0.05). No ocular AEs.
Arriola [63]	Single-center, retrospective, observational	DME/nAMD	100 (85)		In treatment-naïve eyes, BCVA (95% CI) remained stable from 0.4 (0.05 to 0.1) at baseline to 0.6 (0.04 to 0.9) at 4 months (*p* = 0.152). CMT was significantly reduced from 350 μm at baseline to 150 μm at 4 months (*p* < 0.005). In previously treated eyes, BCVA (95% CI) remained stable from 0.3 (0.05 to 0.1) at baseline to 0.3 (0.05 to 0.9) at 4 months (*p* = 0.257). CMT (95% CI) was significantly reduced from 436.5 (225 to 883) μm at baseline to 281 (168 to 587) μm at 4 months (*p* < 0.001). No serious AEs.

AEs: adverse events; BCVA: best-corrected visual acuity; CFT: central foveal thickness; CI: confidence interval; CMT: central macular thickness; CST: central subfield thickness; DME: diabetic macular edema; ETDRS: early treatment diabetic retinopathy study; HRF: hyperreflective foci; IQR: interquartile range; IRF: intraretinal fluid; MAR: minimum angle of resolution; nAMD: neovascular age-related macular degeneration; SD: standard deviation; SRF: subretinal fluid; VA: visual acuity. HRF (Hyperreflective Foci) is believed to be associated with retinal inflammation and may predict a poor visual prognosis in DME) [47].

## Data Availability

No new data were created or analyzed in this study. Data sharing is not applicable to this article.

## Abbreviations

The following abbreviations are used in this manuscript:

AE	Adverse Event
Ang2	Angiopoietin-2
BCVA	Best-Corrected Visual Acuity
CFT	Central Foveal Thickness
CI	Confidence Interval
CMT	Central Macular Thickness
CRT	Central Retinal Thickness
CST	Central Subfield Thickness
DME	Diabetic Macular Edema
EMA	European Medicines Agency
ETDRS	Early Treatment Diabetic Retinopathy Study
FDA	Food and Drug Administration
HRF	Hyperreflective Foci
IOI	Intraocular Inflammation
IQR	Interquartile Range
IRF	Intraretinal Fluid
IVI	Intravitreal injection
MAR	Minimum Angle of Resolution
MBR	Mean Blur Rate
nAMD	Neovascular Age-Related Macular Degeneration
nrDME	Non-responding Diabetic Macular Edema
PCV	Polypoidal Choroidal Vasculopathy
PED	Pigment Epithelial Detachment
PTI	Personalized Treatment Interval
Q8W	Every 8 weeks
Q12W	Every 12 weeks
Q16W	Every 16 weeks
rDME	Responding Diabetic Macular Edema
SD	Standard Deviation
SRF	Subretinal Fluid
T&E	Treat-and-Extend
VA	Visual Acuity
VEGF	Vascular Endothelial Growth Factor
